# Annotations

**Published:** 1907-11-02

**Authors:** 


### ANNOTATIONS.

### The Spread of Disease by Living Agents.

The most commanding feature in recent medical history is, without question, the increase in our knowledge of the causes of disease. Vague surmises about humours, miasmas and the like have yielded all along the line to the definite demonstration of exact and concrete agents. In many instances, as is well recognised, these have been proved to be living organisms, so that the term parasitism has come to have a very widely extended scope. At first the bacteriologist seemed to hold the key to all the etiological positions. More recently, as has been effectively pointed out by Mr. Jas Cantlie, it has been noted that various forms of protozoa occupy a prominent position among the causative agents of disease, and the need for the scientific study of these has been specially realised in the curriculum of the various schools of tropical medicine. Not less important than the living forms which originate so many of the diseases to which human beings are liable is the part played by various animals in the distribution of these pernicious agents. From such gross and obvious examples of transmission as hydatids, glanders, trichinosis, etc., knowledge has gradually extended to a recognition of the part played by animals in the conveyance of tubercle, malaria, filaria, yellow fever, and plague, not to mention sleeping sickness and other less well-known tropical diseases. It may well be questioned whether a still wider range of activity in this direction may not have to be recognised. The common house-fly is already under definite suspicion, and in view of recent investigations into the extension of plague, Sir Lauder Brunton has suggested that in the humble but active flea there may possibly exist a danger to national existence. Certainly the terrible figures of plague mortality in India may well prompt the question whether adequate measures have been taken to prevent the extension of the disease to this country.

### The Proof of Live-Birth.

The question of live-birth is one of the standing difficulties of medico-legal practice. What the law demands as a necessary preliminary to a charge of infanticide—namely, evidence that the child, prior to its death, had been fully delivered from the body of its mother—can rarely be provided by the facts of post-mortem examination. The condition of the lungs may, indeed, show that respiration has taken place; but this fact, apart from other evidence, does not permit an unqualified statement that live-birth, in the legal sense of the term, had certainly taken place. Again, it is customary to suggest that the appearances of natural pulmonary respiration may possibly be produced by artificial inflation of the lungs, even though it is allowed that in the circumstances in which the suspicion of infanticide usually arises any such proceeding is very unlikely to have taken place. This latter proposition is usually discussed in reference to a possible inflation of the lungs by the direct introduction of air into the pulmonary passages through the child's mouth and nostrils. Whatever may be true on this point in any individual instance, it seems certain, from some cases recently recorded by Dr. J. A. Braxton Hicks in the *Westminster Hospital Gazette*, that the general doctrine that the appearances of natural respiration may be exactly simulated by the application of artificial respiration to the body of a still-born child must be accepted as true. Dr. Hicks reports three such cases. In two of these both Silvester's method and Schultze's swinging were practised, whilst in the third mouth-to-mouth respiration was adopted. In each instance the lungs on post-mortem examination had all the appearances of live-birth and gave a positive result to the hydrostatic test. Further, the stomach and upper part of the intestine also contained air, and floated when placed in water, thus providing what has been called the "second live-birth test." From these facts it is evident that a presumptive conclusion in favour of live-birth, based upon the post-mortem appearances of the lungs and stomach, can hardly be upheld when serious though unavailing efforts have been made to establish respiration by artificial methods.

### The Ophthalmological Society.

In his inaugural address as President of this Society, Mr. Marcus Gunn presented an effective and striking summary of the principal work accomplished by the Society since its institution in 1880. The record is a most impressive one, and certainly no professional organisation has more reason to be proud of its achievements. Perhaps the feature which is most worthy of attention is the wide and penetrating character of the subjects which have here been brought under the influence of organised and corporate investigation. Sometimes it is suggested that the so-called specialists have necessarily a confined and narrow outlook, and that they are apt to isolate themselves from the larger currents of professional thought and movement. Granted that such a tendency may exist, it is only fair to recognise that it is often successfully resisted. And this may certainly be said of those who, during the last quarter of a century, have in succession directed the affairs of the Ophthalmological Society. For whilst, most appropriately, the story of the Society includes discussions on such purely surgical matters as Sclerotomy and the Operative Treatment of High Myopia, it also comprises contributions which have a vital relation to the clinical problems of general medical practice. And many of these have become of widespread, or even of universal, value. Take, for example, the study of Eye Symptoms in Locomotor Ataxia by Dr. Hughlings Jackson, or the discussion of the relation between Optic Neuritis and Intracranial Disease introduced by the same authority. The lessons taught in these are now familiar to every senior medical student. No society or work needs a better defence than this, and Mr. Marcus Gunn has, by his historical survey, enforced the debt which general medicine owes to the serious and earnest students of ophthalmology. To some this survey will make it still more a matter for regret that the Ophthalmological Society has not decided to contribute its activities to the larger organisation of the Royal Society of Medicine.

